# Gcn5p and Ubp8p Affect Protein Ubiquitylation and Cell Proliferation by Altering the Fermentative/Respiratory Flux Balance in *Saccharomyces cerevisiae*

**DOI:** 10.1128/mBio.01504-20

**Published:** 2020-08-11

**Authors:** Antonella De Palma, Giulia Fanelli, Elisabetta Cretella, Veronica De Luca, Raffaele Antonio Palladino, Valentina Panzeri, Valentina Roffia, Michele Saliola, Pierluigi Mauri, Patrizia Filetici

**Affiliations:** a Proteomics and Metabolomics Unit, Institute for Biomedical Technologies (ITB-CNR), Segrate, Italy; b Institute of Molecular Biology and Pathology/CNR, Sapienza University of Rome, Rome, Italy; c Department of Biology and Biotechnologies Charles Darwin, Sapienza University of Rome, Rome, Italy; d Institute of Life Sciences, School of Advanced Studies Sant’Anna, Pisa, Italy; Korea Advanced Institute of Science and Technology

**Keywords:** ubiquitylation, Gcn5p, Ubp8p, glycolytic flux, sugar utilization

## Abstract

Protein ubiquitylation regulates not only endocellular trafficking and proteasomal degradation but also the catalytic activity of enzymes. In Saccharomyces cerevisiae, we analyzed the composition of the ubiquitylated proteomes in strains lacking acetyltransferase Gcn5p, Ub-protease Ubp8p, or both to understand their involvement in the regulation of protein ubiquitylation. We analyzed His6Ub proteins with a proteomic approach coupling micro-liquid chromatography and tandem mass spectrometry (μLC-MS/MS) in *gcn5*Δ, *ubp8*Δ and *ubp8*Δ *gcn5*Δ strains. The Ub-proteome altered in the absence of Gcn5p, Ubp8p, or both was characterized, showing that 43% of the proteins was shared in all strains, suggesting their functional relationship. Remarkably, all major glycolytic enzymes showed increased ubiquitylation. Phosphofructokinase 1, the key enzyme of glycolytic flux, showed a higher and altered pattern of ubiquitylation in *gcn5*Δ and *ubp8*Δ strains. Severe defects of growth in poor sugar and altered glucose consumption confirmed a direct role of Gcn5p and Ubp8p in affecting the REDOX balance of the cell.

## Abstract (precis)

We propose a study showing a novel role of Gcn5p and Ubp8p in the process of ubiquitylation of the yeast proteome which includes main glycolytic enzymes. Interestingly, in the absence of Gcn5p and Ubp8p glucose consumption and redox balance were altered in yeast. We believe that these results and the role of Gcn5p and Ubp8p in sugar metabolism might open new perspectives of research leading to novel protocols for counteracting the enhanced glycolysis in tumors.

## INTRODUCTION

Conservation in evolution allows the translation of discoveries obtained in simple models to human cells. Accordingly, simple budding yeast is one of the best models to dissect molecular mechanisms regulating complex networks and circuitries (1). Proteins are differentially regulated by a plethora of posttranslational modifications (PTMs) often involved in a reciprocal cross talk. Lysine acetylation, for example, prevents successive ubiquitylation on the same residue with a direct impact on several functions, including protein turnover and proteasomal degradation (2). The large multiproteic SAGA (Spt-Ada-Gcn5-acetyltransferase) complex is an epigenetic machinery acting through acetylation to induce the transcriptional competence by opening chromatin (3). It is composed of 21 widely conserved proteins grouped into functional submodules, carrying K-acetyltransferase Gcn5p and Ub-protease Ubp8p for deubiquitylation (4–6): this coexistence suggests their interlaced functions. The role of the SAGA complex is not confined to histones but extends to deposition of PTMs on nonhistone proteins. Examples include the interaction of transcription factor IID and the SAGA complex shown by mass spectrometry (7) and the role of Gcn5p and Esa1p in the regulation of cell growth, as shown by SILAC analysis (8). In SAGA, Ubp8p deubiquitylates both histones and nonhistone proteins. For example, deubiquitylase effects on Snf1p’s kinase activity are well established (9), along with the effects of PTMs on the catalytic activity of other enzymes with key roles in energy metabolism (10). We have recently demonstrated that Gcn5p and Ubp8p are involved in the control of respiratory metabolism and mitochondrial functions (11–13).

In the present study, we wanted to analyze whether the global process of protein ubiquitylation might be dependent on Gcn5p, Ubp8p, or both. We performed a shotgun proteomic study of yeast strains disrupted in *GCN5*, *UBP8*, or both, expressing His6-Ub for biochemical purification. Next, we analyzed the composition of Ub proteins with a gel- and label-free proteomic approach based on the coupling of micro-liquid chromatography and tandem mass spectrometry (μLC-MS/MS). We have obtained the proteome composition of His6-Ub proteins and their differential expression in the disrupted strains in comparison with the wild type (WT). By subgrouping the Ub proteins identified as differentially expressed for biological function, we found all the main enzymes involved in glycolysis overrepresented in the absence of Gcn5p and Ubp8p and the key glycolytic enzyme phosphofructokinase 1 (Pfk1pmyc) showed increased and altered ubiquitylation in the disrupted strains. We have also found defective growth in low sugar and altered utilization of glucose with effects on the redox balance of the cell in the absence of Gcn5p and Ubp8p. Collectively, our data indicate that Gcn5p and Ubp8p affect the levels of ubiquitylation of fundamental enzymes and alter the fermentative/respiratory flux balance.

## RESULTS

### Purification of polyubiquitylated proteins in WT, *ubp8*Δ, *gcn5*Δ, and *ubp8*Δ *gcn5*Δ strains.

**FIG 1:**
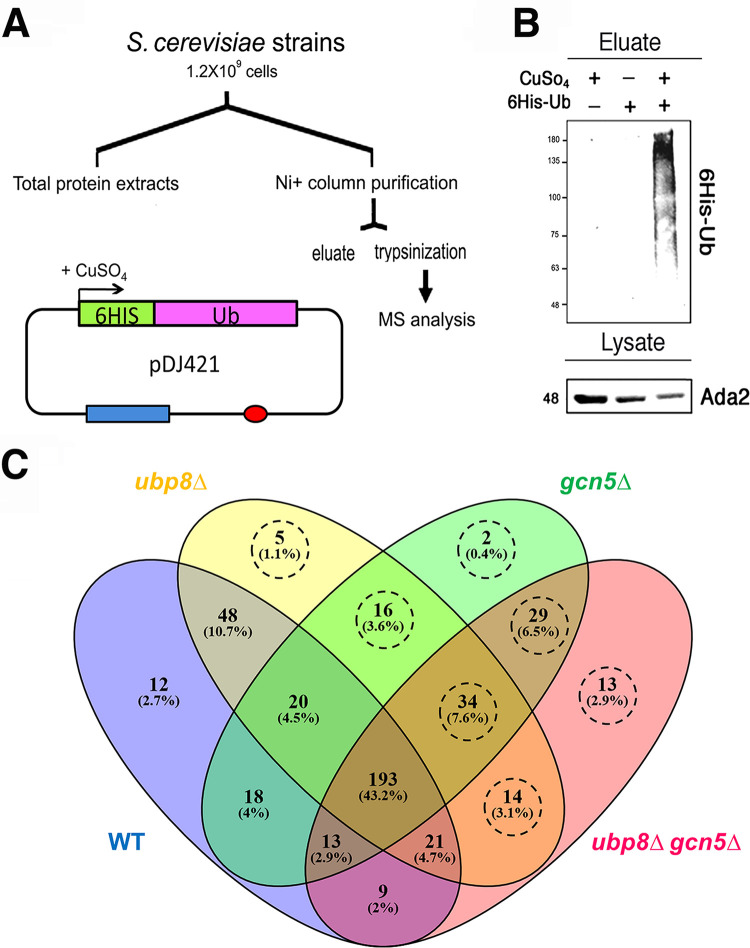
Expression and purification of His6-Ub proteins in S. cerevisiae. (A) Schematic protocol for the expression of His6Ub proteins in strains containing pDJ421 (41). Red oval, origin of replication; blue rectangle, pCUP promoter (LEU cassette). His6-Ub was expressed in CuSO_4_, purified through an Ni^+^ column, and analyzed by MS after trypsinization. (B) Western blot analysis showing the eluate of His6-Ub proteins with respect to the controls hybridized with anti-His6 antibody. Total lysates were probed with anti-Ada2p antibody as an internal standard. (C) Venn diagram of Ub protein distributions found in WT (blue), *ubp8*Δ (yellow), *gcn5*Δ (green), and *ubp8*Δ *gcn5*Δ (pink) strains. The areas of intersection contain proteins common to different strains. The 113 proteins absent in the WT are highlighted by a dashed circle.

The 6×His-Ub proteins were expressed in WT, *ubp8*Δ, *gcn5*Δ, and *ubp8*Δ *gcn5*Δ strains (14), purified on an Ni column, and analyzed by Western blotting (Fig. 1A). Figure 1B shows the selective purification obtained, evidenced with anti-His6 antibody. Total protein extracts (input) were hybridized with anti-Ada2p antibody as an internal loading control. We then performed a proteomic characterization after trypsin digestion of retained 6×His-Ub proteins by a shotgun label-free platform. A total of 16 μLC-MS/MS runs were acquired, evaluating two preparations for the four experimental conditions and analyzing each of them in duplicate. The complete list of proteins identified is reported in Table S1 in the supplemental material. In particular, our proteomic approach allowed the identification of 447 distinct proteins, whose distribution in WT, *gcn5*Δ, *ubp8*Δ, and *ubp8*Δ *gcn5*Δ strains is represented by a Venn diagram (Fig. 1C). Of the 447 identified proteins, 322 Ub proteins were found in common with the WT strain, suggesting that they might change the levels of ubiquitylation and/or expression in the different strains and represent target of *GCN5*, *UBP8*, or both without, however, being considered exclusive substrates. In contrast, the ubiquitylation of 113/447 proteins (circled in dashed lines, Fig. 1C) were considered potential targets of *GCN5* and *UBP8* since they are missing in the wild type. In fact, it should be considered that the deletion may have increased the ubiquitination levels but also modified the overall expression levels of some proteins. Of note, the majority of proteins identified in *ubp8*Δ and *gcn5*Δ strains were also found in the *ubp8*Δ *gcn5*Δ double-mutant strain. A total of 48 proteins have been identified in common between WT/*ubp8*Δ and 18 WT/*gcn5*Δ, suggesting that the lack of Ubp8p is more effective than the lack of Gcn5p (10.7% versus 4%). This observation is not surprising based on the intrinsic deubiquitylase activity of Ubp8p, whereas we expect that Gcn5p acts indirectly, possibly through the deposition of a counteracting acetyl group on lysines.

10.1128/mBio.01504-20.2 TABLE S1 Complete list of Ub proteins detected in WT, *ubp8*Δ, *gcn5*Δ, and *ubp8*Δ *gcn5*Δ S. cerevisiae strains. Download Table S1, XLS file, 0.1 MB. Copyright © 2020 De Palma et al. 2020 De Palma et al. This content is distributed under the terms of the Creative Commons Attribution 4.0 International license.

### Label-free differential analysis.

**FIG 2:**
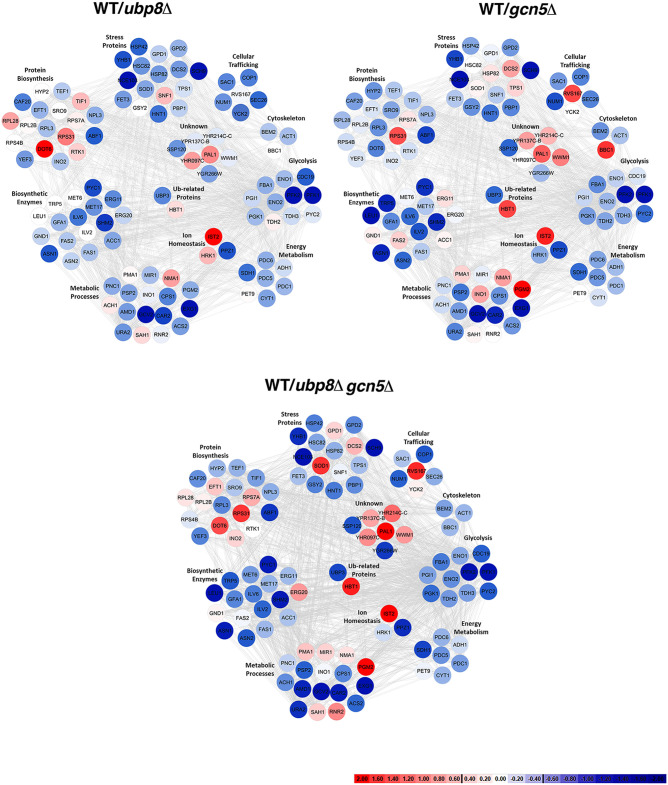
Interactome networks built using STRING database through the mapping of the 103 differentially expressed Ub proteins identified comparing *ubp8*Δ, *gcn5*Δ, and *ubp8*Δ *gcn5*Δ strains versus the WT condition. The color code of distinct nodes represents the DAve value and the relevant chromatic scale (reported in the figure) and ranges from −2.00 to 0 (dark blue to white) and from 0 to +2.00 (white to dark red). Proteins with DAve (ratio of protein expression) ≥ l0.4l and a DCI (confidence of differential expression) ≥ l5l pass the filters and could be considered differentially expressed in the considered comparison. Positive DAve values indicate proteins down-expressed in mutant strains, while negative DAve values indicate proteins up-expressed in mutant strains. The differentially expressed proteins resulting from the three most interesting pairwise comparisons (WT/*ubp8*Δ, WT/*gcn5*Δ, and WT/*ubp8*Δ and *gcn5*Δ) have been plotted on a protein-protein interaction network built by means of STRING database (https://string-db.org) (16). Experimentally and computationally predicted interactions were considered for network construction, setting a confidence score of 0.4. Proteins are represented as colored nodes based on their DAve value and highlighted by gray edges; protein-protein interactions are clustered with respect to the functional pathway.

Using Multidimensional Algorithm Protein Map (MAProMa) software (15), we aligned the 16 protein lists, obtained from biological and technical runs, based on identified proteins and related spectral count values (SpC), which represent the number of spectra attributed to them and indirectly their abundance in the samples. Then, for each strain a unique list was created averaging the spectral count values (SpC*) of the proteins identified with high confidence and with at least one unique peptide. Considering these parameters, MAProMa software estimated the relative protein abundance in the pairwise comparisons of *ubp8*Δ, *gcn5*Δ, and *ubp8*Δ *gcn5*Δ strains respect to the WT strain. To this end, the two algorithms of the software, the differential average (DAve) and the differential confidence index (DCI), were applied, representing the ratio and the confidence in differential expression, respectively, for each protein between two strains. Using stringent filters for DAve and DCI to maximize the confidence of identification and to consider proteins with a variation greater than a fold change of 1.5, a total of 103 proteins were found differentially expressed and reported in Table 1 with selected details and in Table S1 in extended form. In particular, 8 and 49 proteins were, respectively, down- and upregulated in the *ubp8*Δ strain compared to the WT. In addition, 49 proteins were more abundant in the *gcn5*Δ strain compared to WT, and 11 were down-expressed in the *gcn5*Δ strain. Finally, 13 and 65 proteins were down- and up-expressed, respectively, in the *ubp8*Δ *gcn5*Δ strain compared to the WT. In addition, known genetic (G) or physical (P) interactions of each protein with Ubp8p or Gcn5p are indicated in Table 1 with the corresponding human orthologue genes. The trend of the abundance of proteins among the pairwise comparisons of the three mutant strains with respect to the WT is shown through a color code corresponding to the individual DAve values assigned and therefore either to the greater or to the lesser quantity in the mutants than in the WT strains. For positive DAve values, indicating a protein up-expressed in the WT and ranging from +0.40 to +2.00, color gradations are used from light red to dark red and for negative values, indicating a protein up-expressed in mutant strains and ranging from −2.00 to −0.40, chromatic shades from dark blue to light blue. Moreover, in order to give a more comprehensive view of the pathways and of the proteins involved with Gcn5p and Ubp8p interactions, a color code in Table 1 is assigned also to proteins whose DAve and DCI values do not pass the filters in all of the considered comparisons. Proteins with DAve values ranging from −0.4 to +0.4 are indicated with light red and blue, respectively, and unidentified or unchanged ones are indicated in white. Therefore, observing the table and the trends of the differentially expressed proteins among the three comparisons, it is immediately evident that some proteins seem to be more ubiquitylated in the absence of Ubp8p than in the absence of Gcn5p, indicating their major regulatory role on the specific protein. For example, asparagine synthetase (Asn2p), leucine (Leu1p), pyruvate carboxylase (Pyc2p), glyceraldehyde-3-phosphate dehydrogenase (Tdh2p), glycogen synthase (Gsy29p), phosphoglucomutase (Pgm2p), and transposon Ty1-PR1 (Ty1B-PR1p) are proteins that change equally in *gcn5*Δ and double-mutant strains, which suggests that they are functionally related to Gcn5p. In the same way, elongation factor 3A (Yef3p), pyruvate kinase (Cdc19p), cytochrome *c*_1_ (Cyt1p), cytoplasmic chaperone (Hsc82p), and protein involved in clathrin-mediated endocytosis (YHR097Cp) equally change in the *ubp8*Δ and double-mutant strains, thus demonstrating that they are closely related to Ubp8p. Another interesting aspect concerns proteins, such as elongation factor (Eft1p), phosphoglucose isomerase (Pgi1p), NAD-dependent glycerol-3-phosphate dehydrogenase (Gpd1p), superoxide dismutase (Sod1p), AMP deaminase (Amd1p), and uracil (Ura2p), that show a marked increase or reduction in double-mutant *ubp8*Δ *gcn5*Δ strain compared to single-mutant strains, demonstrating the synthetic effect exerted by the double deletion. Finally, in Table 1 we can also observe other proteins, e.g., acetolactate synthase (Ilv6p), phosphofructokinases (Pfk1p and Pfk2p), protein kinase (Sch9p), adenosine 5′-monophosphoramidase (Hnt1p), and glycine decarboxylase (Gcv2p), that show the same differential trend both in single- and double-mutant strains. Noteworthy, the physical (P) or genetic (G) interactions of Ub proteins with either Gcn5p and Ubp8p support these results, indicating a direct effect of Gcn5p and Ubp8p in their modification. Among these, we found proteins that can be grouped on the basis of their functional role, such as amino acid biosynthesis, glycolysis, fermentation, oxidative phosphorylation, and energy metabolism. To better visualize the pathways and the biological relevant interactions, the STRING database (16) was queried to build a network of both known and predicted protein-protein interactions. The protein-protein interaction network (PIN) is a useful tool for systematic investigation and in-depth exploration of biological process mechanisms. Proteins that were differentially expressed were grouped into 10 subnetworks based on their molecular function and are represented by nodes and gray edges indicating protein-protein interactions. Figure 2 shows the three networks obtained with the same chromatic scale of Table 1 and pairwise comparisons of *gcn5*Δ, *ubp8*Δ, and *ubp8*Δ *gcn5*Δ strains against the WT.

**TABLE 1:**
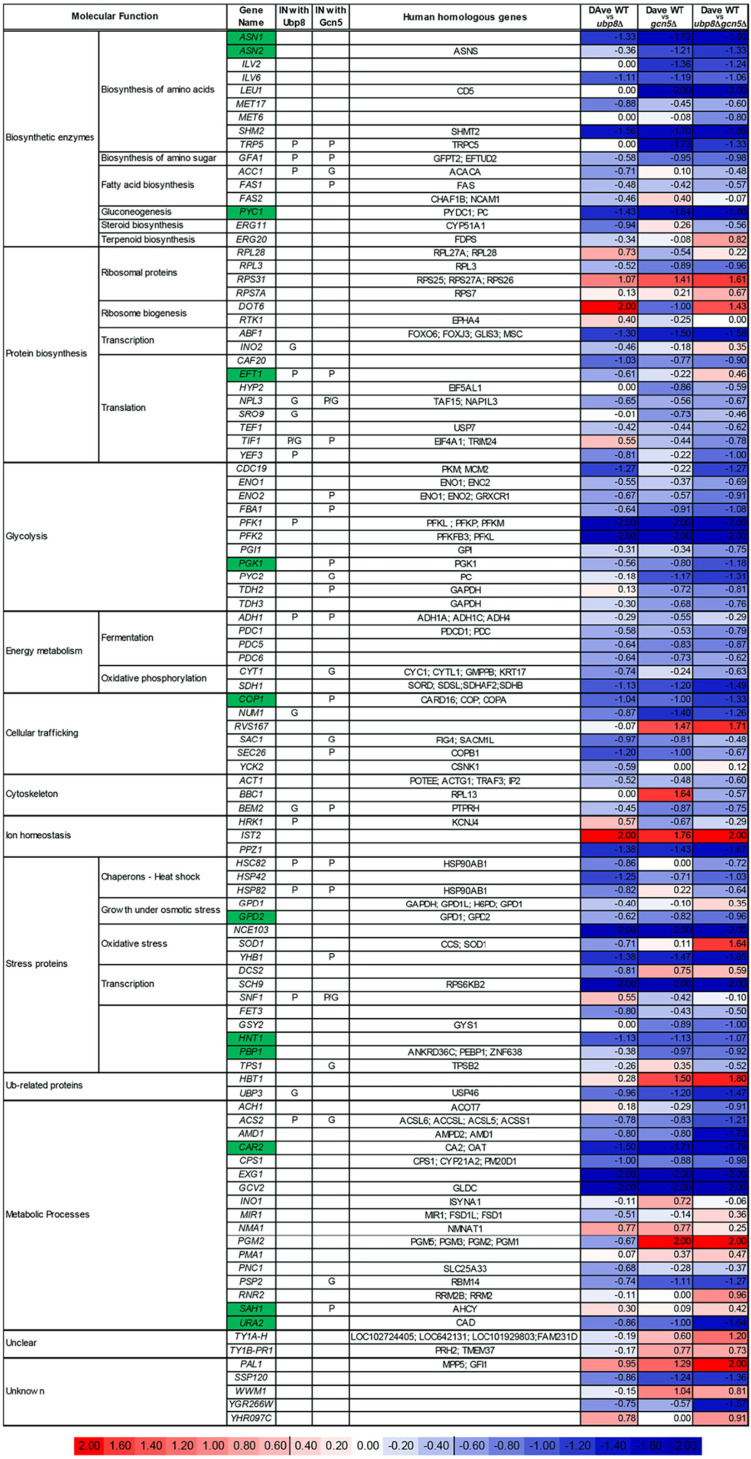
Significant changes in *S. cerevisiae* Ub-proteome of the three mutant strains, as determined by proteomic analysis^*a*^

a Differentially expressed proteins resulted from the MAProMa comparison of *ubp8*Δ, *gcn5*Δ, and *ubp8*Δ *gcn5*Δ strains versus the WT. In particular, each protein is marked by a color code, which is defined by the DAve value obtained in the three examined comparisons. The color is assigned according to a chromatic scale representing the confidence ranges of DAve values adopted (−2.00 to 0 [from blue to white] and +2.00 to 0 [from red to white]). Positive DAve values indicate proteins down-expressed in mutant strains, while negative DAve values indicate proteins up-expressed in mutant strains. The molecular function, gene name, known physical (P) and/or genetic (G) interactions with Ubp8p and/or Gcn5p, and the name of the human orthologues are indicated. Yeast genes corresponding to human pathologies are indicated in green. The complete list of the reported proteins was extracted from the differential lists shown in Table S2 in the supplemental material.

10.1128/mBio.01504-20.3 TABLE S2 Complete list of differentially expressed Ub proteins resulted from the MAProMa comparison of *ubp8*Δ, *gcn5*Δ, and *ubp8*Δ *gcn5*Δ strains versus the WT condition. Download Table S2, XLS file, 0.1 MB. Copyright © 2020 De Palma et al. 2020 De Palma et al. This content is distributed under the terms of the Creative Commons Attribution 4.0 International license.

### Major glycolytic enzymes are differentially ubiquitylated in the absence of Gcn5p or Ubp8p.

**FIG 3:**
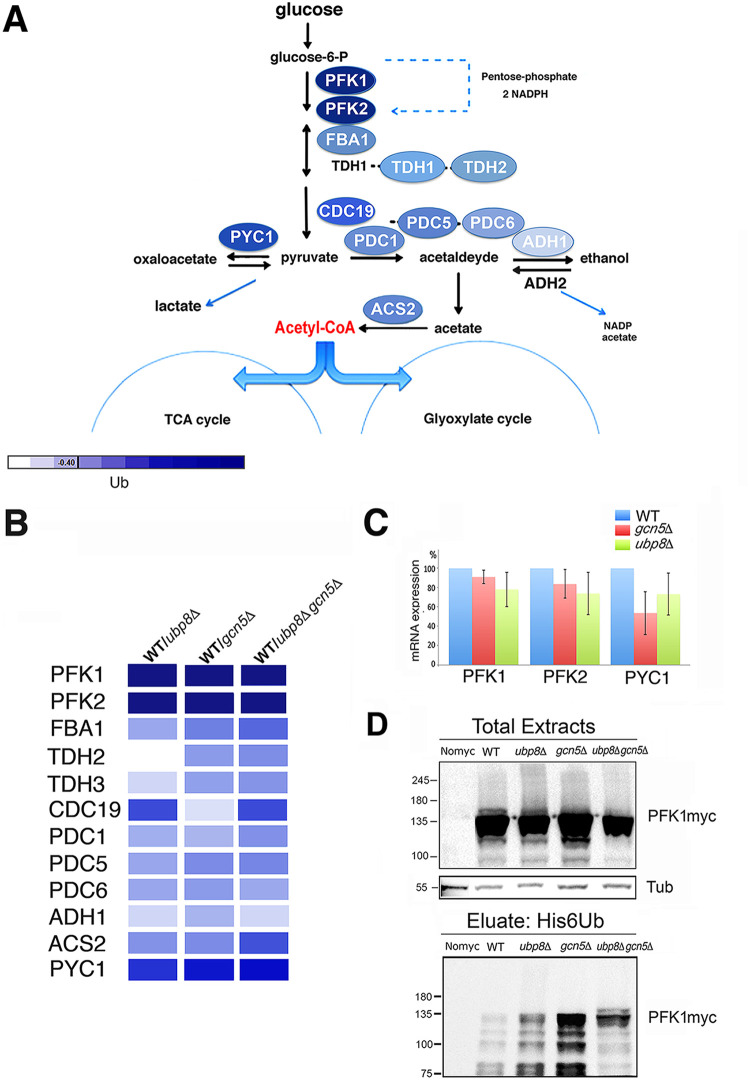
Major glycolytic enzymes are differentially ubiquitylated in the absence of Gcn5p and Ubp8p. (A) Glycolytic pathway in S. cerevisiae. Enzymes are indicated according to the ubiquitylation color code in Fig. 2B. The color code palette found in *ubp8*Δ, *gcn5*Δ, and *ubp8*Δ *gcn5*Δ strains is compared to WT for the indicated enzymes. (C) RT-qPCR of *PFK1*, *PFK2*, and *PYC1* mRNA expression respect to actin in WT, *ubp8*Δ, and *gcn5*Δ strains. (D) Ubiquitylation pattern of Pfk1p the essential enzyme required for initiation of glycolytic flux. His6Ub-Pfk1pmyc version (∼120 kDa) was analyzed by Western blotting in the indicated strains hybridized with anti-myc and anti-tubulin (55 kDa) as internal loading control. The lower panel shows the eluted profiles of His6Ub-Pfk1pmyc in different strains.

Our results show that several altered proteins found in our screening are linked to glycolysis, oxidative stress, and energy metabolism (Fig. 2), supporting the hypothesis that glycolysis might be affected in cells lacking Gcn5p and Ubp8p. The fact that many glycolytic enzymes are ubiquitylated was previously reported by Tripodi et al. (10) in support of a regulatory role of ubiquitylation on these enzymes. Figure 3A shows the major steps of glycolytic flux leading to the production of pyruvate from glucose-6-P. Interestingly, major enzymes involved in these metabolic reactions were identified in our screening and are shown with the color code previously described and presented in yellow in Table S2. This finding suggests an effect of ubiquitylation on glycolysis with possible alterations of glucose consumption. In Fig. 3B, a detailed color palette of identified enzymes is presented showing enhanced ubiquitylation in strains disrupted in Gcn5p, Ubp8p, or both with respect to the WT. Meanwhile, we carried out an analysis of phosphofructokinase (*PFK1* and *PFK2*) and pyruvate carboxylase (*PYC1*) mRNA expression (Fig. 3C). Diagrammed results of RT-qPCR show no effects, excluding those on transcription and confirming that the higher ubiquitylation is at the posttranslational level. We then tested the ubiquitylation pattern of Pfk1p, the essential enzyme required for the initiation of glycolytic flux tagged with myc (Fig. 3D) in strains expressing His6-Ub plasmid. We first analyzed the pattern of total protein extracts with anti-myc antibody, finding enriched signals at high molecular weights (>120 kDa). Then, we obtained proteolytic bands at lower molecular weights (<120 kDa) in agreement with previous reports of proteolytic cleavage of Pfk1p (17). We also found that the intensity of the myc patterns was similar among total protein extracts of strains with respect to a tubulin control. After elution of His6Ub-proteins, in contrast, Ub-Pfk1pmyc showed strong enrichment in the pattern of *ubp8*Δ and *gcn5*Δ strains respect to the wild type. In addition, in the double-mutant *ubp8*Δ *gcn5*Δ strain we found a pattern particularly enriched of higher-molecular-weight bands. This result suggests a synthetic effect in the *ubp8*Δ *gcn5*Δ strain on the ubiquitylation pattern of Pfk1pmyc, indicating the functional interaction between Gcn5p and Ubp8p in this modification. Collectively, these data show that several enzymes necessary for the glycolytic flux are differentially ubiquitylated in the absence of Gcn5p and Ubp8p.

### Glycolysis is impaired in the absence of Gcn5p and Ubp8p.

**FIG 4:**
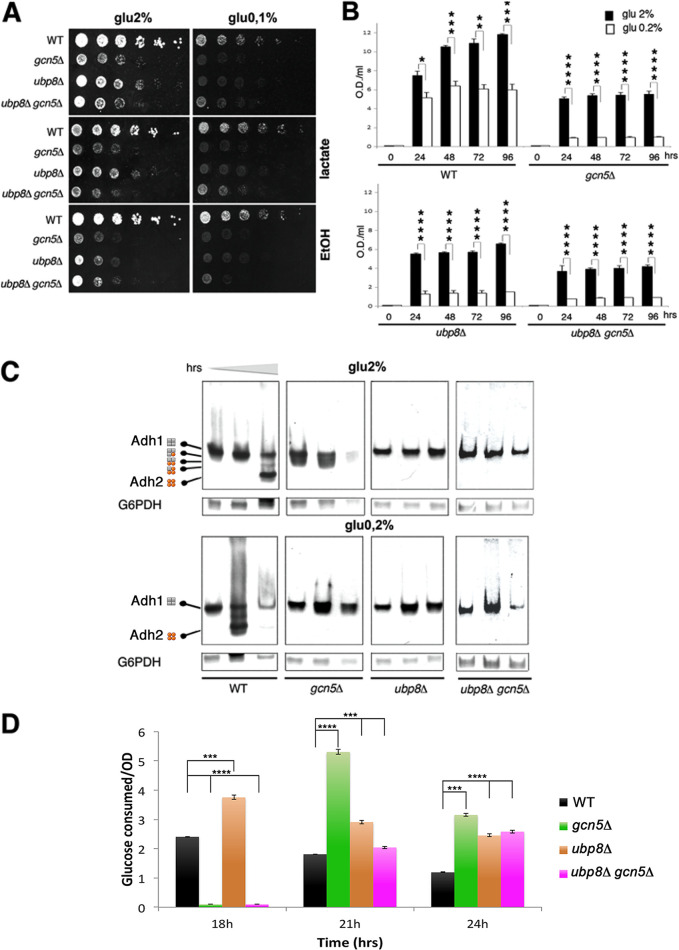
Loss of Gcn5p and Ubp8p causes defects in glycolysis with poor growth in low sugar. (A) Serial dilutions (1:10) of the indicated strains grown for several days on medium containing high (2%) and low (0.1%) glucose show defects in the absence of Gcn5p, Ubp8p, and both not rescued by the addition of lactate and ethanol. (B) Growth of liquid cultures in 2% (black) and 0.1% glucose (white) of WT, *ubp8*Δ *gcn5*Δ, and *ubp8*Δ *gcn5*Δ strains. (C) Alcohol dehydrogenase activities of *ADH1* and *ADH2* stained by a native in-gel assay in the indicated strains grown in 2 and 0.2% glucose for 30, 60, and 90 h, respectively. G6PDHp staining shown as an internal standard. Schematics on the left show the compositions of Adh1p (gray) and Adh2p (orange) homo- and heterotetramers (44). (D) Glucose consumption (g) per 1 OD_600_ of cells of indicated strains.

To determine the impact of *GCN5* and *UBP8* on sugar utilization, wild-type, *gcn5*Δ, *ubp8*Δ, and *gcn5*Δ *ubp8*Δ strains were grown in medium containing high (2%) and low (0.1%) glucose. As shown in Fig. 4A, the three disrupted strains displayed highly reduced growth on 2% glucose plates, and on 0.1% glucose there was a considerable worsening of growth. According to the very poor growth observed on 0.1% glucose, the addition of downstream products such as lactate, ethanol (Fig. 4A), and acetate (not shown) did not ameliorate growth, thus indicating a strong impairment. Mass spectrometry analysis was performed in exponentially growing cells to obtain a picture of ubiquitylation in actively replicating cells of all strains. Then, based on the finding of many enzymes involved in sugar metabolism differentially ubiquitylated in the absence of Gcn5p and Ubp8p, we determined growth curves (see Fig. S1 in the supplemental material) and glucose consumption measurements in liquid cultures of different strains using YP medium and 2 and 0.2% glucose in order to improve the poorer growth obtained in a more stringent condition (0.1% glucose) and under vigorous shaking for optimal oxygenation. In order to evaluate differences among strains, cells were harvested at later intervals of time in order to obtain optimal glucose measurements and highlight metabolic defects in sugar utilization (Fig. 4B). The results confirmed the reduced growth of *gcn5*Δ and *ubp8*Δ strains in high glucose (2%) with respect to the WT, indicating defects in fermentation, that was worse in the *ubp8*Δ *gcn5*Δ strain, reflecting its functional interaction. These results also indicated the greater growth reduction at low sugar levels in all strains, thus confirming the strong delay of growth in poor sugar medium. Collectively, these data demonstrate respiratory and fermentative defects. In particular, the results obtained in liquid culture (Fig. 4B) show a clear synthetic defect of *ubp8*Δ *gcn5*Δ strain with respect to the single disrupted strains and the wild type. This suggests that their functional interaction leads to alteration of the glycolytic flux and redox balance. We used Adh1p and Adh2p, two highly identical alcohol dehydrogenase (ADH) activities, as markers to monitor the channeling of the glucose glycolytic flux toward respiration or fermentation, respectively. In fact, Adh1p is preferentially expressed in fermentative conditions and directly involved in ethanol production (18). In contrast, Adh2p is activated when glucose is exhausted, and the cell switches to a respiratory metabolism and mainly grows by using the accumulated ethanol. Alcohol dehydrogenase activities were assessed by a native in-gel staining method (Fig. 4C) (19–21). The transition from fermentation to respiration can be estimated using an ADH-specific assay (22) previously used to dissect the ADH genetic system (19–21): first, Adh1p followed in fermentation, followed by the appearance of Adh2p, expressed when sugar is exhausted and the cells enter into respiratory metabolism. Protein extracts from cultures of WT, *ubp8*Δ, *gcn5Δ*, and *ubp8*Δ *gcn5*Δ strains grown for 30, 60, and 90 h were subjected to native PAGE and stained for ADH (Fig. 4C) (23). These time course points were again used to follow the metabolic switch in media with high or low sugar. The exclusive presence of Adh1p in the exponential phase indicated fermentative growth, while the progressive appearance of an Adh2p-stained band in 2% glucose (90 h) indicated sugar exhaustion and the switch to respiratory metabolism by ethanol oxidation. In 0.2% glucose, earlier and strong activation of Adh2p occurs at 60 h and highly reduced levels of both Adh1p and Adh2p occur at 90 h due to the exhaustion of available carbon sources (Fig. 4C). The contemporary expression of *ADH1* and *ADH2* leads to the assembly of Adh1p and Adh2p as heterotetramers. Therefore, the very small amount of Adh2p present in all phases of *gcn5*Δ cultures in 2% glucose, as shown in the outlined scheme of Fig. 4C, suggested the putative activation of an intermediate respirofermentative metabolism (24). Conversely, *ubp8*Δ and *ubp8*Δ *gcn5*Δ extracts only displayed Adh1p staining, indicating no activation of respiratory metabolism. In agreement with the growth shown in Fig. 4A and B, the absence of Adh2p in all strains confirmed a strong inability to grow in 0.2% glucose and in the respiratory metabolism, according to previous reports (11, 12). Our collected experimental data demonstrate that, with the disruption of *GCN5*, *UBP8*, or both, cells are unable to adapt their metabolism during the progression from fermentative to respiratory conditions. In order to provide an unbiased demonstration that Ubp8p and Gcn5p are necessary for physiological glycolytic flux progression, we determined the growth curves of WT, *gcn5*Δ, *ubp8*Δ, and *ubp8*Δ *gcn5*Δ strains in 2% glucose medium (see Fig. S1 in the supplemental material) and measured glucose consumption at various time intervals (Fig. 4D). Based on the defective growth of mutant strains, we decided to express the glucose consumption (in g/liter) with respect to cells (optical density at 600 nm [OD_600_]) at 18, 21, and 24 h of growth, and the collected data are shown in a histogram (Fig. 4D). The different glucose consumption behaviors of the three mutant strains are immediately evident if compared to that of the WT control, indicating an inefficient glucose utilization that is not supported by the equivalent cell growth capabilities (Fig. S1).

10.1128/mBio.01504-20.1 FIG S1 Growth curves (filled circles) of indicated strains in high glucose (2%) are diagrammed with measures (open circles) of glucose (in g/liter) consumed at increasing times (in h) in the same strains. Download FIG S1, PDF file, 0.5 MB. Copyright © 2020 De Palma et al. 2020 De Palma et al. This content is distributed under the terms of the Creative Commons Attribution 4.0 International license.

## DISCUSSION

**FIG 5:**
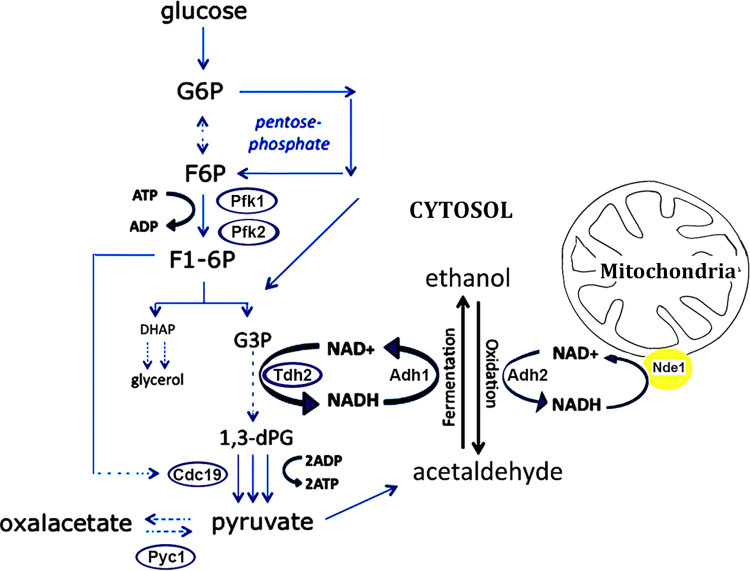
Metabolic effects on glycolysis in the absence of Gcn5p and Ubp8p. The model shows enzymes whose ubiquitylation is altered in the absence of Gcn5p and Ubp8p and possible routes redirecting glycolytic flux toward fermentation, gluconeogenesis/anaplerotic routes, or a pentose phosphate shunt according to the altered redox balance of the mutants. The partial reoxidation of the cytosolic NADH by the mitochondrial transdehydrogenase Nde1p in *gcn5Δ* is deduced by the contemporary presence of Adh1p and Adh2p in high glucose medium.

A complex code of posttranslational modifications is responsible for the dynamic regulation of chromatin and modifications of histone and nonhistone proteins. Gcn5p was first described as a pleiotropic, transcriptional coactivator involved in the acetylation of histones (25, 26), and it soon gained importance as a prototype of chromatin modifiers required in chromatin remodeling and gene activation. Its central and pleiotropic role was further highlighted by evidences showing that acetylation and phosphorylation were found engaged in the regulation of the cell cycle and growth rate in yeast (27, 28). Ubp8p, another subunit of the SAGA complex, was also described in the process of histone H2B deubiquitylation, contributing with histone acetylation to the opening of chromatin and active transcription (6, 29) and in other processes, such as the regulation of chromosome segregation through modification of the centromeric histone variant Cse4p (14). However, while the roles of these modifiers in different chromatin-related functions have been widely described (30), their role in the modulation of nonhistone proteins and their effects on protein stability, cellular localization, and enzymatic activity have been far less investigated. In yeast, gene expression cannot explain the metabolic changes of the cell in response to sudden changes of the environment. Accordingly, Tripodi et al. reported that acetylation and ubiquitylation marked nodal enzymes involved in the control of sugar metabolism, in particular glycolysis (10), and suggested the role of acetylation and ubiquitylation in the metabolic response and signaling to biochemical pathways. The present investigation was undertaken to gain insights into the effects of SAGA components on the ubiquitylation of total proteins. We carried out a comparison of the Ub proteins identified by a proteomic analysis in strains disrupted in *GCN5*, *UBP8*, or both in comparison to the wild type. We used the μLC-MS/MS system for the first characterization of H6-Ub proteins obtained from the four examined strains, and then we obtained quantitative information on specific proteins belonging to cellular metabolic processes subjected to fast changes in response to genetic or environmental stimuli. In this way, we were able to identify the trend of relative abundance of differentially expressed proteins among the three mutated strains compared to the WT. In addition, based on proteomic results, we drafted a proteomic interaction map and a grouping of protein subclasses with respect to their biological function for a better visualization of the major pathways involved. In the first instance, the results obtained clearly indicate a central role for Ubp8p and Gcn5p, affecting specific proteins that contribute to the control of cell growth, stress response, and—above all—energetic metabolism. Most of the Ub proteins detected as differentially expressed in the examined comparisons, although not always with the same levels of abundance, were shared and uprepresented in the absence of *GCN5* and *UBP8* and in the double-disrupted strain. These evidences can be interpreted as due to modification of lysines that can alternatively be acetylated or ubiquitylated (10) or even to a lower expression of *UBP8* in *gcn5*Δ strain (12). As also reported by Wilson et al. (9), our data confirmed that SAGA modulates the PTMs of Snf1p, is involved in the inactivation of enzymes of fatty acid biosynthesis and glycogen storage, and is a positive regulator of autophagy (31). Accordingly, a lower ubiquitylation of this protein in the *ubp8*Δ strain was detected in our analysis. It has to be mentioned that the biochemical purification of His6-Ub proteins carried out in our study is used for the study of nonhistone proteins and not for chromatin; therefore, it is not expected for the evaluation of known Ubp8p targets such as histone H2B. The interaction map of Ub proteins grouped in functional categories highlights the presence of all major enzymes involved in glycolysis, thus suggesting a strong impact of the ubiquitylation of these enzymes in the utilization of glucose (Table 2 and Fig. 2; see also Table S2 in the supplemental material). Remarkably, the importance of posttranslational modifications of metabolic proteins in carbon metabolism is highlighted (10). For the identified proteins Pfk1p and Pfk2p, key nodes for the execution of the glycolytic flux (32) showed higher ubiquitylation in the absence of Gcn5p, Ubp8p, or both and no variation at the transcriptional level. We also analyzed the ubiquitylation pattern of Pfk1pmyc and found altered ubiquitylation levels and a synthetic effect in the *ubp8*Δ *gcn5*Δ strain (Fig. 3D) in accordance with the proteomic data. Our results agree with the ubiquitylation contribution to the allosteric regulation of the catalytic activity of Pfk1p and Pfk2p reported in the literature (33). In the present work, we suggest a model for alternative metabolic redox-balancing routes undertaken in the absence of Gcn5p and Ubp8p caused by altered glycolysis. Adh1p and Adh2p activities were used as metabolic markers in a native PAGE staining assay (19, 20, 22, 23) to determine how glucose is metabolized in *gcn5Δ*, *ubp8Δ*, and *ubp8Δ gcn5Δ* strains compared to the wild type. Our results demonstrated that growth was severely impaired in 0.2% glucose in the absence of *GCN5* and *UBP8*. Accordingly, the exclusive presence of Adh1p in the three disrupted strains (Fig. 4C) indicated a fermentative condition and their inability to switch to respiratory metabolism (11, 12). Nonetheless, the *gcn5Δ* strain displayed a very small amount of Adh2p in fermentative conditions (2% glucose), suggesting the contemporary accumulation of ethanol and its partial oxidation by Adh2p with activation of both fermentative and respiratory metabolism. In fact, while Adh1p allows the glycolytic flux to proceed to ethanol through the reoxidation of NADH in the first part of glycolysis, Adh2p oxidizes part of the ethanol to acetaldehyde. This reaction provides the cytoplasmic NADH redox excess for the transdehydrogenases (Nde1p and Nde2p) localized on the outer mitochondrial membrane that transfer these equivalents to the respiratory transport chain (Fig. 5). We cannot exclude the activation of other mechanisms for the oxidation of cytosolic NADH by mitochondria (34). The schematic model (Fig. 5) summarizes alternative metabolic pathways to be undertaken in the absence of *GCN5* and *UBP8* in high glucose. In this respect, we suggest that the differential capacity of external and internal NADH dehydrogenases in the two deleted strains, interpreted in terms of redox metabolism, plays crucial roles in fermentation (24). In low 0.2% glucose, there is a complete lack of Adh2p, the gene for which is usually regulated by Adr1p and repressed in high glucose (35). In addition to Pfk1p and Pfk2p, showing highest ubiquitylation level in the absence of *GCN5* and *UBP8*, other important enzymes, such as Tdh2p, Pyc1p, and Cdc19p at the crossroads of glycolysis, gluconeogenesis, and pentose phosphate pathways, showed altered ubiquitylation in both deletion strains (Fig. 3A and B). These data indicate that *gcn5Δ*, *ubp8Δ*, and *ubp8Δ gcn5Δ* cells lose the controlled metabolic routes and cell proliferation program, as determined by the combined higher levels of ubiquitylation and increased consumption of glucose and, in this context, the increased glycolytic flux, that, like a river, is dispersed into many little streams. The presented results open a novel field of investigation and demonstrate the important role of SAGA components Gcn5p and Ubp8p in affecting the ubiquitylation of important metabolic enzymes involved in sugar utilization. Our results also indicate a reciprocal function of Gcn5p and Ubp8p in coregulating the ubiquitylation process. This is not surprising since lysine acetylation often counteracts further ubiquitylation, suggesting that there is a reciprocal interlaced role of these factors (2). We think that the presented results may open the way for a novel framework of research with interesting implications for the study of cancer cells where increased glucose utilization and altered glycolytic flux occur independently to oxygen availability (36, 37). In case of proinflammatory stimuli, for example, the induction of a metabolic switch leading to upregulation of aerobic glycolysis (38) leads to an enhanced utilization of glucose that sustains the high rate of cell proliferation and invasiveness (39). The role of Gcn5p and Ubp8p affecting protein ubiquitylation in yeast is a novel finding that sheds light on the importance of the epigenetic posttranslational regulation not only for nucleosome remodeling but also at the metabolic level. In summary, we show a direct requirement of SAGA acetyltransferase Gcn5p and Ub-protease Ubp8p in the regulation of this process, strengthening previous data that indicated the presence of acetylated and ubiquitylated versions among glycolytic enzymes (10). As a possible translational application of our results, we recall the role of the “cancer signature gene” Usp22, an orthologue of Ubp8p, in aggressive tumors such as kidney tumors and glioma (40). We might therefore envisage novel strategies that may alter energy supply and lower glycolysis by targeting SAGA components as novel approaches to slow proliferation and invasion of cancer cells.

**TABLE 2:** Saccharomyces cerevisiae strains derived from isogenic W303

Strain	Genotype	Source or reference
W303	*MAT***a** *ade2-1 trp1-1 leu2-3112 his3-11*,*15 ura3 can1-100ssd*	27
YPO4	*MAT***a** *ade2-1 trp1-1 leu2-3112 his3-11*,*15 ura3 can1-100ssd gcn5*::*KanMX4*	27
YFT21	*MAT***a** *ade2-1 trp1-1 leu2-3112 his3-11*,*15 ura3 can1-100ssd ubp8*::*HIS3MX6*	14
YFT19	*MAT***a** *ade2-1 trp1-1 leu2-3112 his3-11*,*15 ura3 can1-100ssd CSE4*::*Cse4myc12-URA3 ubp8*::*HIS3MX6 gcn5*::*KanMX4*	14
YCM1	*MAT***a** *ade2-1 trp1-1 leu2-3112 his3-11*,*15 ura3 can1-100ssd CSE4*::*Cse4myc12-URA3 + pJD421His6-Ub-LEU*	14
YCM3	*MAT***a** *ade2-1 trp1-1 leu2-3112 his3-11*,*15 ura3 can1-100ssd CSE4*::*Cse4myc12-URA3 ubp8*::*HIS3MX6 + pJD421His6-Ub-LEU*	14
YCM2	*MAT***a** *ade2-1 trp1-1 leu2-3112 his3-11*,*15 ura3 can1-100ssd CSE4*::*Cse4myc12-URA3 gcn5*::*KanMX4 + pJD421His6-Ub-LEU*	This study
YCM4	*MAT***a** *ade2-1 trp1-1 leu2-3112 his3-11*,*15 ura3 can1-100ssd CSE4*::*Cse4myc12-URA3 gcn5*::*KanMX4 ubp8*::*HIS3MX6 + pJD421His6-Ub-LEU*	This study
YVD01	*MAT***a** *ade2-1 trp1-1 leu2-3112 his3-11*,*15 ura3 can1-100ssd PFK1*::*9myc-HIS3MX6*	This study
YVD02	*MAT***a** *ade2-1 trp1-1 leu2-3112 his3-11*,*15 ura3 can1-100ssd gcn5*::*KanMX4 PFK1*::*9myc-HIS3MX6*	This study
YVD03	*MAT***a** *ade2-1 trp1-1 leu2-3112 his3-11*,*15 ura3 can1-100ssd ubp8*::*HIS3MX6 PFK1*::*9myc-klTRP1*	This study
YVD04	*MAT***a** *ade2-1 trp1-1 leu2-3112 his3-11*,*15 ura3 can1-100ssd gcn5*::*KanMX4 ubp8*::*HIS3MX6 PFK1*::*9myc-klTRP1*	This study
YVD05	*MAT***a** *ade2-1 trp1-1 leu2-3112 his3-11*,*15 ura3 can1-100ssd PFK1*::*9myc-HIS3MX6+ pJD421His6-Ub-LEU*	This study
YVD06	*MAT***a** *ade2-1 trp1-1 leu2-3112 his3-11*,*15 ura3 can1-100ssd gcn5*::*KanMX4 PFK1*::*9myc-HIS3MX6+ pJD421His6-Ub-LEU*	This study
YVD07	*MAT***a** *ade2-1 trp1-1*,*leu2-3112*,*his3-11*,*15 ura3 can1-100ssd*, *ubp8*::*HIS3MX6*, *PFK1*:: *9myc-klTRP1+ pJD421His6-Ub-LEU*	This study
YVD08	*MAT***a** *ade2-1 trp1-1 leu2-3112 his3-11*,*15 ura3 can1-100ssd gcn5*::*KanMX4 ubp8*::*HIS3MX6 PFK1*::*9myc-klTRP1+ pJD421His6-Ub-LEU*	This study

## MATERIALS AND METHODS

### Yeast strains and growth.

The Saccharomyces cerevisiae strains examined here derive from isogenic strain W303 and are listed in Table 2. Gene disruptions and protein myc tagging were controlled by colony PCR and Western blotting. His6-Ub was expressed from pDJ421 plasmid with pCUP promoter (41). Growth was performed at 28°C in YP medium (1% yeast extract, 2% Bacto peptone, 1% agar) containing 2, 0.2, or 0.1% glucose and 3% glycerol, along with 1% ethanol, 1% lactate, or 1% acetate. For the growth assay, 1 OD_600_ of exponentially growing cells was serially diluted (1:10) and spotted.

### Expression, protein extraction, and purification of His6-Ub proteins.

For total protein extraction, yeast cultures were collected at exponential phase (0.8 OD_600_/ml), and pellets were treated with 0.3 M NaOH–140 mM β-mercaptoethanol and then incubated in ice with occasional vortexing. Trichloroacetic acid (TCA; 55%) was added, followed by incubation in ice. After centrifugation, the cells were lysed in HU buffer (8 M urea, 5% sodium dodecyl sulfate [SDS], 200 mM Tris [pH 6.8], 0.1 mM EDTA, 100 mM dithiothreitol, bromophenol blue) and boiled 15 min at 65°C. Cells transformed with pDJ421 encoding 6×His-Ub (41) were grown on selective media and copper-inducible promoter activated overnight with 0.1 mM CuSO_4_. Cells (0.8 to 1 OD_600_) were then lysed in guanidinium buffer (6 M guanidinium hydrochloride, 0.1 M Na_2_HPO_4_/NaH_2_PO_4_, 0.01 M Tris-HCl [pH 8.0], 0.1% Triton X-100, 5 mM imidazole, 10 mM β-mercaptoethanol, 0.005 M NEM, 0.1 mM MG132 and protease inhibitors); 1/20th of lysate was used as input and TCA (5.5%) precipitated. The lysate was then incubated on a Ni-NTA agarose resin (Qiagen) at 4°C with rotation, after washing with urea buffer (8 M urea, 0.1 M Na_2_HPO_4_/Na_2_HPO_4_, 0.01 M Tris-HCl [pH 6.4], 3.5 mM β-mercaptoethanol, and 0.1% Triton X-100), the samples were eluted in 2× sample buffer (0.125 M Tris-HCl [pH 6.8], 4% SDS, 0.285 M β-mercaptoethanol, 2% bromophenol blue, 4 ml of 100% glycerol). For MudPIT analysis, the resin was washed three times with 8 M urea buffer and with 1 M urea buffer; trypsin was then added in 1:20 (wt/wt) enzyme-substrate. After overnight digestion (37°C), the reaction was stopped by acidification with 1 μl of trifluoroacetic acid.

### SDS-PAGE, Western blotting, and immunohistochemistry.

Yeast cultures were grown at 28°C semicomplete medium (0.67% yeast nitrogen base, 2% glucose, 0.1% dropout mix). For expression of His6-Ub, 0.1 mM CuSO_4_ was added to the cultures (Iglesias). Cells were collected (0.8 OD_600_/ml), and the eluate and lysate samples were loaded on 7.5% SDS-PAGE, blotted on nitrocellulose membranes (Amersham), and hybridized with anti-6×His-Ub (Abcam), anti-Ada2p, anti-myc, and anti-α-tubulin (Santa Cruz). Proteins were detected by using Long-Lasting Chemilumiscent substrate (EuroClone) and visualized by the ChemiDoc MP imaging system (Bio-Rad).

### Real-time qRT-PCR.

Exponentially growing cells (WT, *gcn5*Δ, and *ubp8*Δ strains) in YP plus 2% glucose were collected. Total RNA extraction was performed using the phenol method and retrotranscribed with a QuantiTect reverse transcription kit (Qiagen). Standard curves of WT genomic DNA (20/0.05 ng) and cDNA (50 ng) were amplified for actin, phosphofructokinases (*PFK1* and *PFK2*), and pyruvate carboxylase (*PYC1*); all experiments were performed in triplicate. Quantitative RT-PCR (qRT-PCR) experiments were carried out on a Rotor-gene Q (Qiagen) apparatus. The following oligonucleotides that were used: ACT1f, 5′-GCTGAAAGAGAAATTGTCCG-3′; ACT1r, 5′-ACACTTCATGATGGAGTTGTA-3′; PFK1f, 5′-GCTCAAAGTCAAGGTGCTCT-3′; PFK1r, 5′-CCTGAGAAGGTGATGTTGTTG-3′; PFK2f, 5′-GCAGTTTCAACCAAGCCAAC-3′; PFK2r, 5′-CGTTAGAGTTCATACCTGGAG-3′; PYC1f, 5′-CGTACCGCTCATGAACTGT-3′; and PYC1r, 5′-GGATGGTGAAATCTACCTGG-3′.

### Protein extraction, estimation, and tryptic digestion for proteomic analysis.

Tryptic digest mixtures were desalted using Pierce C_18_ spin columns (Thermo Fisher Scientific) and resuspended in 0.1% formic acid. Samples were then analyzed by means of a μLC system coupled with a tandem mass spectrometer through a trap-elute configuration. Briefly, 5 μl (3 μg) of each digested peptide mixture was first loaded onto a peptide trap (Zorbax 300 SB-C18, 0.3 i.d. by 5 mm, 5 μm, 300 Å; Agilent Technologies, Santa Clara, CA) for concentration and desalting with a pump running in isocratic mode with 0.1% formic acid in water. Then, the automatic switching of a 10-port valve eluted the trapped mixture on a reversed-phase column (Biobasic-C18, 0.180 i.d., 100- mm length, 5-μm particle size; Thermo Fisher Scientific) for the separation with an acetonitrile gradient (eluent A, 0.1% formic acid in water; eluent B, 0.1% formic acid in acetonitrile); the gradient profile was 5% eluent B for 5 min, followed by 5 to 40% eluent B for 45 min, 40 to 80% eluent B for 10 min, 80 to 95% eluent B for 5 min, and 95% eluent B for 10 min. The flow rate was 100 μl/min, which was split to achieve a final flux of 2 μl/min. The peptides that were eluted from the C_18_ column were analyzed directly with a linear ion trap LTQ mass spectrometer equipped with a nano-ESI source (Thermo Fisher Scientific). The spray capillary voltage was set at 1.6 kV, and the ion transfer capillary temperature was held at 220°C. Full mass spectra were recorded over a 400- to 2,000-*m/z* range in positive-ion mode, followed by five tandem mass spectrometry (MS/MS) events sequentially generated in a data-dependent manner on the top five most intense ions selected from the full MS spectrum, using a dynamic exclusion for MS/MS analysis. In particular, MS/MS scans were acquired setting a normalized collision energy of 35% on the precursor ion. MS scan functions and LC solvent gradients were controlled by the Xcalibur data system, version 1.4 (Thermo Fisher Scientific). A total of 16 LC-MS/MS runs were performed, representing the four yeast strain conditions examined (WT, *ubp8*Δ, *gcn5*Δ, and *ubp8*Δ *gcn5*Δ) as technical and biological replicates.

### Mass spectrometry data handling and database search.

All data generated were searched using the Sequest HT search engine contained in the Thermo Scientific Proteome Discoverer software (v2.1) against the UniProt S. cerevisiae proteome database. The following criteria were applied: trypsin as enzyme, three missed cleavages per peptide, mass tolerances of ± 0.8 Da for precursor ions and ± 0.6 Da for fragment ions, protein grouping, and strict parsimony principle. Percolator node was used with a target-decoy strategy to give final false discovery rates at a peptide spectrum match level of 0.01 (strict), considering a maximum delta CN of 0.05 (42). Only peptides with high confidence, a minimum peptide length of six amino acids, and rank 1 were considered. The output data obtained from Discoverer software consist of identified protein lists for each sample analyzed, accompanied by information related to them, including spectral counts (SpCs), i.e., the total number of spectra attributed to each protein. The Euler diagrams were calculated using Venny 2.1 drawing tool (software at http://bioinfogp.cnb.csic.es/tools/venny/) using the UniProt accession numbers. Cellular function was assigned to each protein according to the GOA database (http://geneontology.org/) and the UniProt database (http://www.uniprot.org/). We were unable to assign the cellular function for some proteins, which were therefore classified as “unknown,” and additional proteins without a complete characterization at the moment of data analysis were classified as “unclear” or “other.” For label-free differential analysis, the 16 protein lists obtained from the SEQUEST algorithm were aligned with an in-house algorithm, MAProMA (Multidimensional Algorithm Protein Map) (15) and compared by means of the average spectral counts (aSpCs) corresponding to the average of all the spectra identified for a protein and, consequently, to its relative abundance, in each analyzed condition (WT, *ubp8*Δ, *gcn5*Δ, and *ubp8*Δ *gcn5*Δ). In depth, to select differentially expressed proteins, the four subgroups were compared pairwise, applying thresholds of 0.4 and 5 on the two MAProMa indexes DAve (differential average) and DCI (differential confidence index), respectively. DAve, which evaluates changes in protein expression, was defined as (*X – Y*)/(*X + Y*)/0.5 with values ranging from a maximum of + 2.00 to a minimum of −2.00, while DCI, that evaluates the confidence of differential expression, was defined as (*X + Y*) × (*X – Y*)/2 with values ranging from +∞ to –∞. The *X* and *Y* terms represent the aSpCs of a given protein in two compared samples. Most confident up-represented proteins showed a DAve ≥ +0.4 and a DCI ≥ +5; most confident down-represented proteins showed a DAve ≤ −0.4 and DCI ≤ −5.

### In-gel staining and glucose measurements.

Cell extracts preparation, native polyacrylamide gel electrophoresis (PAGE), alcohol dehydrogenase (Adhp), and glucose-6-phosphate 1-dehydrogenase (G6PDHp) staining assays were carried out as previously described (23, 43). Adh1p and Adh2p protein amounts have been determined from native PAGE with the program ImageJ. Glucose concentration in the culture supernatants were measured by using an R-Biopharma kit (Darmstadt, Germany) according to the manufacturer’s instructions and expressed as grams of glucose consumed/OD_600_.

### Statistical analysis.

Experimental results shown in Fig. 4B and C represent the averages of three different biological replicates; the standard errors are also shown. Asterisks represent the statistical significance determined with the Student *t* test (****, *P* < 0.0001; ***, *P* < 0.001; **, *P* < 0.01; *, *P* < 0.05). The error bars shown in Fig. 3C show the standard deviations of two biological replicates.
