# Supplementary figures and images for: Gcn5p and Ubp8p Affect Protein Ubiquitylation and Cell Proliferation by Altering the Fermentative/Respiratory Flux Balance in Saccharomyces cerevisiae
Source: mBio. 2020 Aug 11;11(4):e01504-20. doi: 10.1128/mBio.01504-20 (PMC7439465; doi:10.1128/mBio.01504-20)

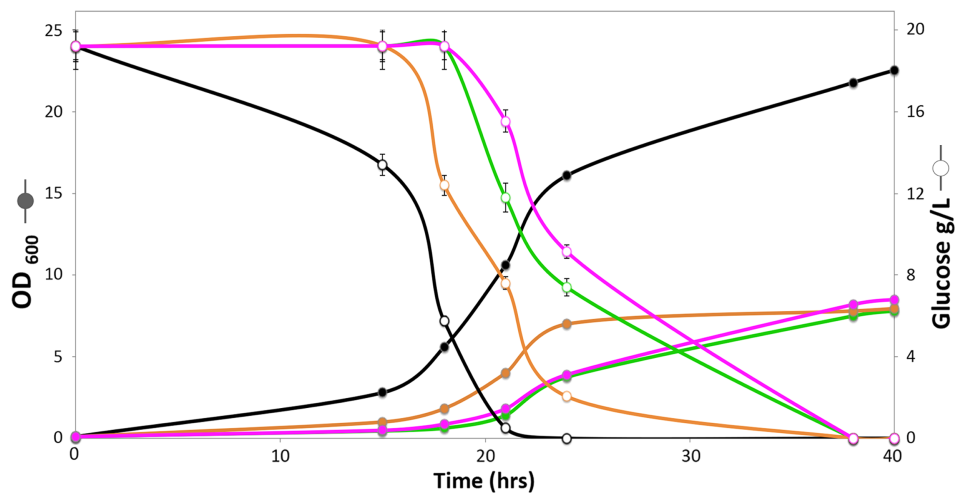

Supplement: FIG S1 [file mBio.01504-20-sf001.pdf]
